# Adipose mTORC2 is essential for sensory innervation in white adipose tissue and whole-body energy homeostasis

**DOI:** 10.1016/j.molmet.2022.101580

**Published:** 2022-08-23

**Authors:** Irina C. Frei, Diana Weissenberger, Danilo Ritz, Wolf Heusermann, Marco Colombi, Mitsugu Shimobayashi, Michael N. Hall

**Affiliations:** Biozentrum, University of Basel, Basel 4056, Switzerland

**Keywords:** Adipose tissue, Whole-body energy homeostasis, mTORC2, Sensory nervous system, Diabetes, CGRP, Neuropathy

## Abstract

**Objective:**

Adipose tissue, via sympathetic and possibly sensory neurons, communicates with the central nervous system (CNS) to mediate energy homeostasis. In contrast to the sympathetic nervous system, the morphology, role and regulation of the sensory nervous system in adipose tissue are poorly characterized.

**Methods and results:**

Taking advantage of recent progress in whole-mount three-dimensional imaging, we identified a network of calcitonin gene-related protein (CGRP)-positive sensory neurons in murine white adipose tissue (WAT). We found that adipose mammalian target of rapamycin complex 2 (mTORC2), a major component of the insulin signaling pathway, is required for arborization of sensory neurons, but not of sympathetic neurons. Time course experiments revealed that adipose mTORC2 is required for maintenance of sensory neurons. Furthermore, loss of sensory innervation in WAT coincided with systemic insulin resistance. Finally, we established that neuronal protein growth-associated protein 43 (GAP43) is a marker for sensory neurons in adipose tissue.

**Conclusion:**

Our findings indicate that adipose mTORC2 is necessary for sensory innervation in WAT. In addition, our results suggest that WAT may affect whole-body energy homeostasis via sensory neurons.

## Highlights

• Whole-mount three-dimensional imaging revealed an extensive sensory neuronal network in white adipose tissue (WAT).
• Adipose mTORC2 is required for arborization of sensory, but not sympathetic neurons in WAT.
• Loss of sensory neurons in WAT coincides with systemic insulin resistance.
• The neuronal protein GAP43 is a marker for sensory innervation in WAT.

## 1 Introduction

Communication between tissues mediates whole-body energy homeostasis [1]. Disruption of this communication causes disorders such as obesity and type 2 diabetes [2]. It is well established that WAT communicates with other tissues, including the brain, by secreting adipokines such as leptin and adiponectin [3,4]. WAT also interacts with the central nervous system (CNS) via sympathetic and sensory neurons [[5], [6], [7]]. The sympathetic nervous system transmits signals from CNS to WAT. For example, fasting triggers sympathetic neurons to release the neurotransmitter norepinephrine (NE) in WAT. NE then stimulates adipocytes to release free fatty acids that provide energy for other tissues [8]. Compared to the sympathetic nervous system, sensory neurons in WAT are less studied. Detection of the sensory neuron markers calcitonin gene-related peptide (CGRP), substance P, and advillin revealed the presence of sensory neurons in WAT [[9], [10], [11], [12]]. Anterograde tracing using herpes simplex virus subsequently confirmed the transmission of sensory information from WAT to CNS [13]. Further studies identified the adipokine leptin and bioactive free fatty acids as potential sensory stimuli in WAT [14,15]. However, the morphology, role and regulation of the sensory nervous system in WAT remain poorly understood.

The mammalian target of rapamycin complex 2 (mTORC2) is a multiprotein serine/threonine kinase. It is composed of four core components including the kinase subunit mTOR and the mTORC2-specific subunit rapamycin-insensitive companion of mTOR (RICTOR) [[16], [17], [18], [19]]. Studies in adipose-specific *Rictor* knockout mice revealed that loss of adipose mTORC2 causes reduced glucose uptake and impaired lipid handling in adipocytes [[20], [21], [22], [23], [24], [25]]. Furthermore, loss of adipose mTORC2 non-cell-autonomously causes hyperinsulinemia and systemic insulin resistance [20,21,23]. These data suggest that adipose mTORC2 is a critical regulator of systemic energy homeostasis. However, the mechanisms by which adipose mTORC2 mediates communication with other tissues are poorly understood.

We used an inducible adipose-specific *Rictor* knockout (iAdRiKO) mouse to study how adipose mTORC2 mediates WAT communication with other tissues. Phosphoproteomic analysis of inguinal WAT (iWAT) from iAdRiKO mice revealed acute changes in phosphorylation of proteins associated with neurons. To study the effect of adipose mTORC2 on neurons, we visualized sympathetic and sensory neuronal networks in iWAT by performing whole-mount imaging. We discovered that arborization of CGRP-positive sensory neurons, but not tyrosine hydroxylase (TH)-positive sympathetic neurons, was diminished in WAT upon loss of adipose mTORC2. Furthermore, we found that the neuronal protein growth-associated protein 43 (GAP43) is expressed specifically in sensory neurons in WAT. We conclude that adipose mTORC2 is required for the stability of sensory neurons in WAT, thereby mediating adipocyte-to-CNS communication.

## 2 Materials and methods

### 2.1 Mice

As an early step in the generation of an iAdRiKO mouse line, *Rictor*^*fl/fl*^ mice were bred with *adipoq-CreER*^*T2*^ mice provided by Stefan Offermanns (Max Planck Institute for Heart and Lung Research [MPI-HLR], Bad Nauheim, Germany) [26,27]. For experiments, *Rictor*^*fl/fl*^ *adipoq-CreER*^*T2*^ mice were bred with *Rictor*^*fl/fl*^ mice to generate iAdRiKO and control littermates lacking the *CreER*^*T2*^ transgene. *Rictor* knockout was induced at 6 weeks of age. For that, iAdRiKO and control littermates were injected intraperitoneally with 1 mg/mouse tamoxifen (Sigma–Aldrich) resuspended in corn oil, for 5 consecutive days. Mice were housed at 22 °C in a regulation compliant facility under a 12-hour light/12-hour dark cycle with unlimited access to water and normal diet (unless stated otherwise). Mice were sacrificed early in the morning and tissues were collected and weighed. All mouse experiments were performed according to federal guidelines for animal experimentation and were approved by the Kantonales Veterinäramt of the Kanton Basel-Stadt under the cantonal license 2602 and 2975.

### 2.2 2-Deoxyglucose uptake assay

Mice were fasted for 5 h, then injected i. p. with Humalog insulin (Lilly; 0.75 U/kg body weight), followed 10 min later with an injection of 2-deoxyglucose (Sigma–Aldrich; 32.8 μg/g body weight). Tissues were collected 20 min after administration of 2-deoxyglucose. Tissues were lysed in 10 mM Tris–HCL, pH 8.0, by boiling for 15 min 2-Deoxyglucose-6-phosphate (2DGP) was measured using a Glucose Uptake-Glo Assay Kit (Promega) following the manufacturer's instructions.

### 2.3 Insulin tolerance test

Mice were fasted for 5 h and blood samples were collected to determine blood glucose levels. Humalog insulin was given i. p. (Lilly; 0.75 U/kg body weight), and blood glucose levels were monitored by an Accu-Chek blood glucose meter for 90 min.

### 2.4 Immunoblots

Tissue were homogenized in lysis buffer containing 100 mM Tris–HCl pH 7.5, 2 mM EDTA, 2 mM EGTA, 150 mM NaCl, 1% Triton X-100, cOmplete inhibitor cocktail (Roche) and PhosSTOP (Roche). Protein concentration was determined by Bradford assay. Equal amounts of protein were separated by SDS-PAGE and transferred onto nitrocellulose membranes (GE Healthcare). The nitrocellulose membranes were blocked with 5% BSA in TBST (TBS containing 0.1% Tween20) and incubated overnight in primary antibody diluted in TBST containing 5% BSA. Primary antibodies used were RICTOR (1:1000; Cell signaling; Cat#2140), AKT (1:1000; Cell signaling, Cat#4685), AKT-pS473 (1:1000; Cell signaling, Cat#9271), CALNEXIN (1:1000, Enzo, Cat#ADI-SPA-860-F), GAP43 (1:1000, Cell signaling, Cat#8945), GAP43-pS41 (1:1000, R&D Systems, Cat#PPS006), Tyrosine hydroxylase (1:500, Millipore, Cat#AB1542), HSL (1:2000, Cell signaling, Cat#4107), HSL-pS660 (1:1000, Cell signaling, Cat#4126), HSL-pS563 (1:1000, Cell signaling, Cat#4139). The primary antibody was washed several times with TBST and then incubated in secondary antibody in TBST containing 5% milk powder (w/v). Secondary antibodies used were mouse anti-rabbit (1:10′000, Jackson, 211-032-171) and rabbit anti-sheep (1:10′000, Invitrogen, 81-8620).

### 2.5 Sample preparation for proteomics and phosphoproteomics

An extended description of sample preparation for proteomics is in Appendix A Supplementary Materials & Methods. In short, tissues were pulverized and homogenized in lysis buffer containing 100 mM Tris–HCl pH7.5, 2 mM EDTA, 2 mM EGTA, 150 mM NaCl, 1% Triton X-100, cOmplete inhibitor cocktail (Roche) and PhosSTOP (Roche). Samples were lysed by polytron followed by ultrasonication. Proteins were precipitated by trichloroacetic acid, alkylated digested with modified trypsin (enzyme/protein ratio 1:50) overnight. Peptides were desalted using C18 reverse-phase spin columns (Macrospin, Harvard Apparatus) according to the manufacturer's instructions, dried under vacuum and stored at −20 °C until further use.

For TMT-labelling, 25 μg of peptides per sample were labeled with isobaric tandem mass tags (TMT10plex, Thermo Fisher Scientific) as described [28].

### 2.6 Proteomics

An extended description of the proteomics procedure and analysis is in Appendix A Supplementary Materials & Methods. TMT-labeled peptides were fractionated by high-pH reversed phase separation using a XBridge Peptide BEH C18 column (3.5 μm, 130 Å, 1 mm × 150 mm, Waters) on an Agilent 1260 Infinity HPLC system as previously described [29] and dried under vacuum. Peptides were subjected to LC–MS/MS analysis using a Q Exactive HF Mass Spectrometer fitted with an EASY-nLC1000 (both Thermo Fisher Scientific). Peptides were resolved using a RP-HPLC column (75 μm × 30 cm) packed in-house with C18 resin, heated to 60 °C. The acquired raw-files were searched using MASCOT against a murine database (consisting of 34026 forward and reverse protein sequences downloaded from Uniprot on 20190129), the six calibration mix proteins [28] and 392 commonly observed contaminants. The database search results were imported into the Scaffold Q+ software (version 4.3.2, Proteome Software Inc., Portland, OR) and the protein false identification rate was set to 1% based on the number of decoy hits. Acquired reporter ion intensities in the experiments were employed for automated quantification and statistical analysis using a modified version of our in-house developed SafeQuant R script [28]. Finally, significantly deregulated proteins were defined as log2 (fold change) > 0.5 or log2 (fold change) < −0.5, p-value < 0.01. We note that not all neuropeptides were detected, due to the limitations of untargeted mass spectrometry.

### 2.7 Phosphoproteomics

An extended description of the phosphoproteomics procedure and analysis are in Appendix A Supplementary Materials & Methods. Peptide samples were enriched for phosphorylated peptides using Fe(III)-IMAC cartridges on an AssayMAP Bravo platform as described [30]. Unmodified peptides (“flowthrough”) were subsequently used for TMT analysis. Phospho-enriched peptides were subjected to LC–MS/MS analysis using an Orbitrap Fusion Lumos Mass Spectrometer fitted with an EASY-nLC 1200 (both Thermo Fisher Scientific). Peptides were resolved using a RP-HPLC column (75 μm × 37 cm) packed in-house with C18 resin heated to 60 °C.The acquired raw-files were imported into the Progenesis QI software (v2.0, Nonlinear Dynamics Limited), which was used to extract peptide precursor ion intensities across all samples applying the default parameters. The generated mgf-file was searched using MASCOT against a murine database (consisting of 34026 forward and reverse protein sequences downloaded from Uniprot on 2019-01-29) and 392 commonly observed contaminants. The database search results were filtered using the ion score to set the false discovery rate (FDR) to 1% on the peptide and protein level. Exported peptide intensities were normalized based on the protein regulations observed in the corresponding TMT experiment in order to account for changes in protein abundance. Only peptides corresponding to proteins, which were regulated significantly with a p value ≤ 1% in the TMT analysis were normalized. Quantitative analysis results from label-free quantification were processed using the SafeQuant R package v.2.3.2 [28] (https://github.com/eahrne/SafeQuant/) to obtain peptide relative abundances. The summarized peptide expression values were used for statistical testing of between condition differentially abundant peptides. All proteins detected are presented in Supplementary Table 2. Significantly deregulated hits were selected by a calculated p-value < 0.01. Finally, statistical analysis for phosphoprotemics was performed using the Software Perseus [31] and pathway enrichment analysis was performed by the Database for Annotation, Visualization and Integrated discovery (DAVID) v6.8 [32].

### 2.8 Immunofluorescent staining of WAT sections

Inguinal WATs were fixed overnight in 4% formalin at room temperature, dehydrated, embedded in paraffin, and cut into 5 μm thick sections. For immunofluorescent staining, sections were rehydrated and antigen retrieved by boiling sections in target retrieval Solution (Dako) for 20 min in a KOS Microwave HistoSTATION (Milestone Medical). Sections were blocked using Protein block serum-free ready-to-use (Dako) and then incubated in primary antibody diluted in antibody diluent with background reducing component (Dako) overnight at 4 °C. Primary antibodies against tyrosine hydroxylase (1:500, Millipore, Cat#AB1542), CGRP (1:200, Enzo, BML-CA1137-0100), GAP43-pS41 (1:200, R&D system, PPS006), Neurofilament heavy polypeptide (1:1000, Abcam, ab8135), Synaptophysin (1:200, Abcam, ab32127) and Adiponectin (1:500, Invitrogen, PA1-054) were used. After washing, sections were incubated in secondary antibody (1:500) in antibody diluent with background reducing component (Dako) for 1 h at room temperature. Secondary antibodies against rabbit (Invitrogen, A21070 or A21443), sheep (Invitrogen, A21436) and mouse (Invitrogen, A11004) were used. Finally, sections were stained with NucBlue Live Cell Staining ReadyProbes reagent (Invitrogen, R37605) for 2 min and mounted with VECTASHIELD (Vector, H-1000). Images were obtained using Applied Precision Deltavision CORE system (Leica) and analyzed with OMERO [33]. For quantification, maximal intensity levels were used.

### 2.9 Whole mount imaging using inguinal WAT depots

Mouse tissues were harvested after intracardiac perfusion with 4% Paraformaldehyde (PFA) and further fixed in 4% PFA overnight at 4 °C.

For immunolabeling, tissue clearing and high-resolution volumetric imaging, we developed a modified protocol with focusing on preservation of tissue structure, antibody compatibility, and fluorescence. First steps were as described [34]. In short, tissues were washed with PBS, dehydrated using a methanol/B1n buffer (0.3 M Glycine, 0.1% (v/v) Triton-X, pH 7), delipidated using dichloromethane, bleached overnight with 5% H2O2 at 4 °C and rehydrated in a reversed methanol/B1n buffer series.

For subsequent immunolabeling, tissues were incubated with primary antibodies against TH (1:500, Millipore, Cat#AB1542) and CGRP (1:750, ImmunoSTAR, Cat#24112) diluted in PTxwH buffer (PBS, 0.1% Triton-X (v/v), 0.05% Tween20 (v/v), 2 μg/ml Heparin), and with secondary antibodies against sheep (1:500, Invitrogen, A21436 or A21448) and/or rabbit (1:500, Invitrogen, A21070 or A21443).

Further tissue clearing was performed using the water-based clearing method Cubic L [35]. Briefly, samples cleared by CUBIC L solution (10% N-butyldiethanolamine (v/v), 10% Triton X-100 (v/v) in ddH_2_0) followed by 2% agarose embedding and refractive index (RI) matched in CUBIC RA solution (45% antipyrine, 30% N-methylnicotinamide) before imaging. After RI matching, tissue was imaged with the Zen black software (ZEISS) in Cubic RA solution with the RI of 1.51 on a Carl ZEISS lightsheet 7 microscope equipped with the Clr Plan-Neofluar 20×/1.0 detection objective and dual side illuminated with 10×/0.2 objectives. Acquired tiles were loaded in ArivisVision4D (Arivis) for stitching, visualization, and analysis. For tracing sensory neurons, nerve fibers were reconstructed in 3D and total neurite length (μm) was determined using the FilamentTracer tool (Imaris, Oxford instruments).

### 2.10 CGRP ELISA

Tissues were lysed in PBS using lysis matrix D tubes (MP Biomedicals). CGRP levels in lysates were determined using mouse calcitonin gene-related peptide (CGRP) ELISA kit (CSB-EQ027706MO, Cusabio) according to the manufacturer's instructions.

### 2.11 Denervation surgery

Inguinal fat depots were denervated in eight-week-old mice as described [36]. For surgical denervation, mice were anaesthetized and incisions were made dorsally on the flank. Nerves innervating inguinal WAT were identified using a dissection microscope, cut several times and removed. For sham operation, mice were anaesthetized and incisions were made dorsally on the flank. Mice were allowed to recover for four weeks after surgery before being sacrificed for further analysis.

### 2.12 Statistics

Sample size was chosen according to our previous studies and published reports in which similar experimental procedures were described. The investigators were not blinded to the treatment groups. All data are shown as the mean ± SD. Sample numbers are indicated in each figure legend. For mouse experiments, *n* represents the number of animals, and for imaging, *N* indicates the number of images acquired per experiment. To determine the statistical significance between two groups, an unpaired two-tailed Student's t-test was performed. For ITT, two-way ANOVA was performed. All statistical analyses were performed using GraphPad Prism 9 (GraphPad Software, San Diego, California). A *p* value of less than 0.05 was considered statistically significant.

## 3 Results

### 3.1 Loss of adipose mTORC2 acutely impairs whole-body energy metabolism

**Figure 1:**
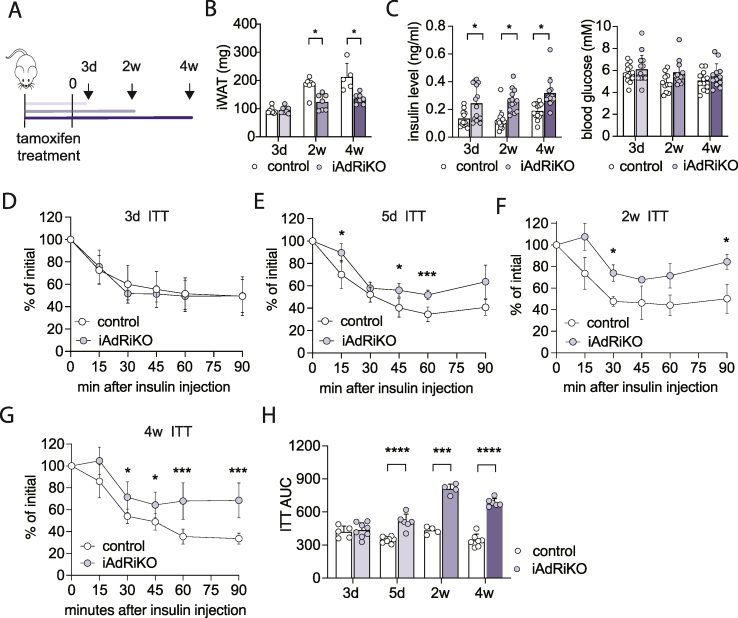
**Loss of adipose mTORC2 acutely impairs whole-body energy homeostasis**. (A) Experimental design of the longitudinal studies. (B) Tissue weight of inguinal WAT (iWAT) in *ad libitum*-fed control and iAdRiKO mice three days (n = 8; 7), two (n = 7), and four weeks (n = 5; 7) after tamoxifen treatment. Student's t-test, ∗p < 0.05. (C) Plasma insulin and blood glucose levels of control and iAdRiKO mice after 16 h starvation three days (n = 13; 15), two (15; 13), and four weeks (13; 12) after tamoxifen treatment. Student's t-test, ∗p < 0.05. (D) Insulin tolerance test (ITT) on control and iAdRiKO mice three days after tamoxifen treatment (n = 5; 8). (E) ITT on control and iAdRiKO mice five days after tamoxifen treatment (n = 8; 6). 2-way ANOVA, ∗p < 0.05, ∗∗∗p < 0.001. (F) ITT on control and iAdRiKO mice two weeks after tamoxifen treatment (n = 4). 2-way ANOVA, ∗p < 0.05. (G) ITT on control and iAdRiKO mice four weeks after tamoxifen treatment (n = 5; 6). 2-way ANOVA, ∗p < 0.05, ∗∗∗p < 0.001. (H) Area under the curve (AUC) for ITT on control and iAdRiKO mice three days (n = 5; 8), five days (n = 8; 6), two (n = 4), and four weeks (n = 5; 6) after tamoxifen treatment. Student's t-test, ∗∗∗p < 0.001, ∗∗∗∗p < 0.0001.

**Supplementary Figure 1:**
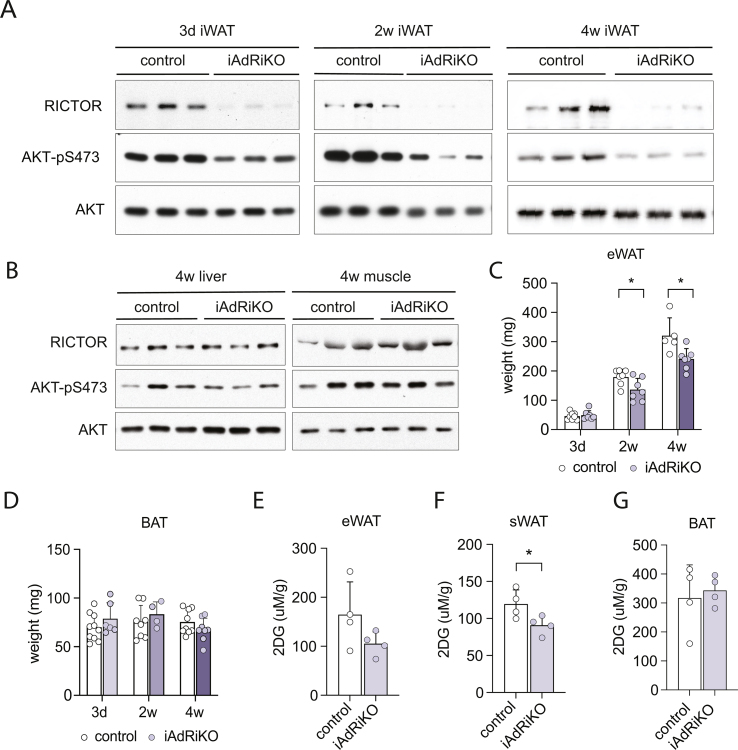
(A) Immunoblot analysis of inguinal WAT (iWAT) from control and iAdRiKO mice three days, two, and four weeks after tamoxifen treatment (n = 6; 6). (B) Immunoblot analysis of liver and muscle tissue from control and iAdRiKO mice four weeks after tamoxifen treatment (n = 6; 6). (C) Tissue weight of epididymal WAT (eWAT) in *ad libidum*-fed control and iAdRiKO mice three days (n = 8; 7), two (n = 7), and four weeks (n = 5; 7) after tamoxifen treatment. Student's t test, ∗p < 0.05. (D) Tissue weight of brown adipose tissue (BAT) in *ad libidum*-fed control and iAdRiKO mice at three days (n = 10; 6), two (n = 8; 4) and four (n = 9; 8) weeks after tamoxifen treatment. (E) 2-deoxyglucose (2DG) uptake in eWAT of control and iAdRiKO mice three days after tamoxifen treatment (n = 4). (F) 2DG uptake in iWAT of control and iAdRiKO mice three days after tamoxifen treatment (n = 4). Student's t-test, ∗p < 0.05. (G) 2DG uptake in BAT of control and iAdRiKO mice three days after tamoxifen treatment (n = 4).

Mice lacking adipose mTORC2 from birth display a defect in whole-body energy homeostasis characterized by hyperinsulinemia and systemic insulin resistance [20,21,23]. Since adipose tissue is not fully developed until six weeks of age, this could be due to a developmental defect. To determine whether and, if so, how soon loss of mTORC2 in mature adipocytes causes systemic effects, we performed a longitudinal study with tamoxifen-inducible *Rictor* knockout mice (iAdRiKO; *Rictor*^*fl/fl*^*Adipoq promoter-CreER*^*T2*^) (Figure 1A). Six week old mice were treated with tamoxifen daily for five days and analyzed three days, two weeks, and four weeks after the last tamoxifen treatment (Figure 1A). *Cre*-negative mice treated with tamoxifen served as controls. As expected, expression of RICTOR and phosphorylation of the mTORC2 target AKT (AKT-pS473) were reduced in WAT of iAdRiKO mice at all three post-tamoxifen timepoints (Figure S1A). In liver and muscle, RICTOR expression and AKT phosphorylation were not affected (Figure S1B). Loss of mTORC2 in mature adipocytes reduced WAT mass approximately 30% compared to control mice (Figures 1B and S1C), while brown adipose tissue (BAT) mass was unchanged (Figure S1D).

Next, we characterized the metabolic state of iAdRiKO and control mice. Fasting insulin levels were increased in iAdRiKO mice already at the first time point (three days) and remained significantly elevated (Figure 1C). Blood glucose levels were normal throughout the time course (Figure 1C). These results suggest that elevated insulin compensates for reduced insulin sensitivity upon loss of mTORC2, as observed previously in mice lacking adipose mTORC2 at birth [20,23]. We note that loss of adipose mTORC2, as expected, reduced glucose uptake in WAT (Figure S1E–G). Furthermore, iAdRiKO mice became insulin resistant, as determined by an insulin tolerance test (Figure 1D–H). Severe systemic insulin resistance was detected in iAdRiKO mice two and four weeks after tamoxifen treatment (Figure 1F–H). Mild insulin resistance was detected in iAdRiKO mice five days, but not three days, post-tamoxifen treatment (Figure 1D-E and H).Thus, loss of adipose mTORC2 leads to acute development of hyperinsulinemia and gradual development of systemic insulin resistance. These results highlight the importance of adipose mTORC2 in whole-body energy homeostasis.

### 3.2 Loss of mTORC2 alters proteins associated with synapse formation in WAT

**Figure 2:**
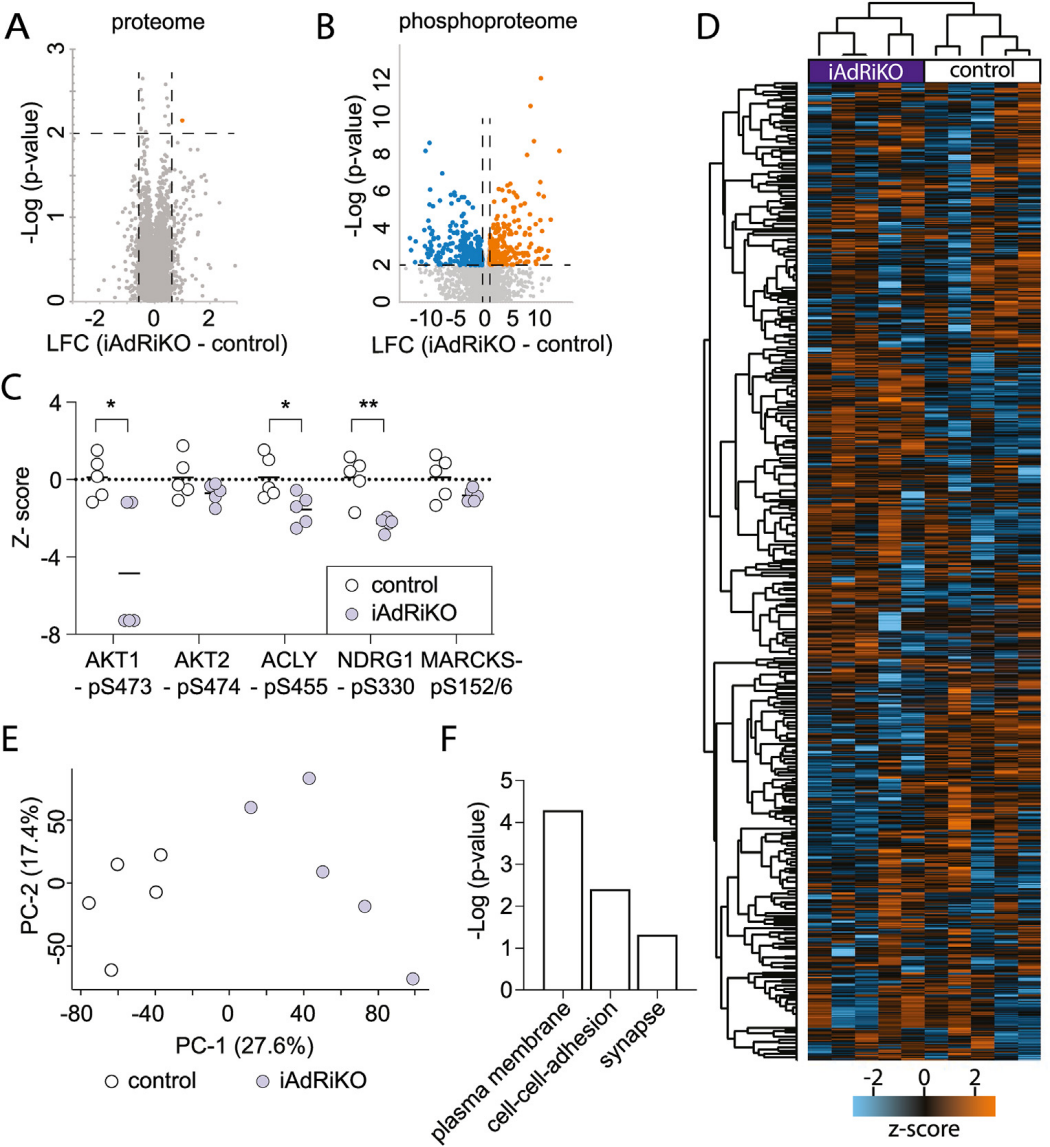
**Loss of mTORC2 affects proteins associated with synapse formation in WAT.** (A) Volcano-plot displaying the comparison of proteins derived from inguinal WAT (iWAT) of control and iAdRiKO mice at three days after tamoxifen treatment (n = 5). LFC = Log_2_ fold change. (B) Volcano-plot displaying the comparison of phosphosites derived from iWAT of control and iAdRiKO mice three days after tamoxifen treatment (n = 5). LFC = Log_2_ fold change. (C) Z-scores of mTORC2-regulated phosphosites in iWAT three days after tamoxifen treatment (n = 5). Student's t-test, ∗p < 0.05, ∗∗p < 0.01. (D) Unsupervised hierarchical clustering using Euclidian distance of the phosphoproteome data set (n = 5). (E) Principal component analysis (PCA) for iWAT-derived phosphoproteome of control and iAdRiKO mice three days after tamoxifen treatment (n = 5). (F) Pathway enrichment analysis of phosphoproteins altered in iWAT of iAdRiKO mice compared to control mice analyzed and visualized by the Database for Annotation, Visualization and Integrated Discovery (DAVID).

To gain better understanding of the role of adipose mTORC2 in whole-body energy homeostasis, we performed proteomic and phosphoproteomic analyses on iWAT obtained from iAdRiKO and control mice three days after tamoxifen treatment. This time point allowed us to study the acute effects of reduced mTORC2 activity (Figure S1A), before development of systemic insulin resistance (Figure 1D–H). The proteomic analysis identified and compared 6′082 proteins in iWAT of iAdRiKO and control mice. Only one protein was significantly altered in iAdRiKO mice three days after tamoxifen treatment (40 S ribosomal protein S24; p < 0.01; |log_2_(iAdRiKO/control)| > 0.6) (Figure 2A; Supplementary Table 1A). In contrast, the phosphoproteomic analysis detected and quantified 10,443 phosphorylated sites of which 319 and 249 were significantly up- and down-regulated (p < 0.01), respectively, upon loss of adipose mTORC2 (Figure 2B; Supplementary Table 1B). As expected, mTORC2 downstream readouts AKT1-pS473, ACLY-pS455 [37], NDRG1-pS330 [38] and MARCKS-pS152/156 [39] were downregulated in iWAT of iAdRiKO mice compared to control iWAT (Figure 2C). Next, we examined whether loss of mTORC2 in adipocytes elicits a molecular signature. Indeed, unsupervised hierarchical clustering analysis (Figure 2D) and principal component analysis (PCA) (Figure 2E) of the phosphoproteomic data identified two distinct clusters corresponding to iAdRiKO mice and control mice.

**Supplementary Figure 2:**
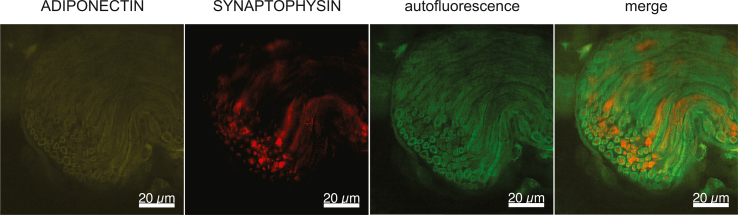
Representative image of large nerve bundles in control iWAT immunostained with both ADIPONECTIN and SYNAOTIPHYSIN (N = 4). Scale bar = 20 μm.

To identify molecular processes associated with loss of mTORC2 signaling, we performed pathway enrichment analysis. Proteins whose phosphorylation was significantly altered were associated with the plasma membrane and cell–cell adhesion (Figure 2F). This observation is in agreement with previous studies showing that many mTORC2 substrates are localized to the plasma membrane [40,41]. Unexpectedly, the pathway enrichment analysis also revealed changes in proteins associated with the nervous system, in particular synapse-associated proteins (Figure 2F). To investigate the possibility of ectopic *Adipoq promoter-CreER*^T2^ expression and thus mTORC2 ablation in neurons, we examined adiponectin expression in neurons in iWAT. We found no evidence for adiponectin expression in nerve bundles innervating iWAT (Figure S2). This finding is supported by large-scale single-cell RNA sequence data on mouse dorsal root ganglia[42], where iWAT-innervating sensory neurons originate. Thus, changes in phosphorylation of synapse-associated proteins are unlikely due to ectopic ablation of mTORC2 in neurons. Thus, we hypothesize that loss of adipose mTORC2 impacts the neuronal network in iWAT. Furthermore, our findings suggest that the acute effects of mTORC2 loss are due to changes in protein phosphorylation rather than expression, as expected for loss of a kinase.

### 3.3 Loss of adipose mTORC2 reduces arborization of sensory neurons

**Figure 3:**
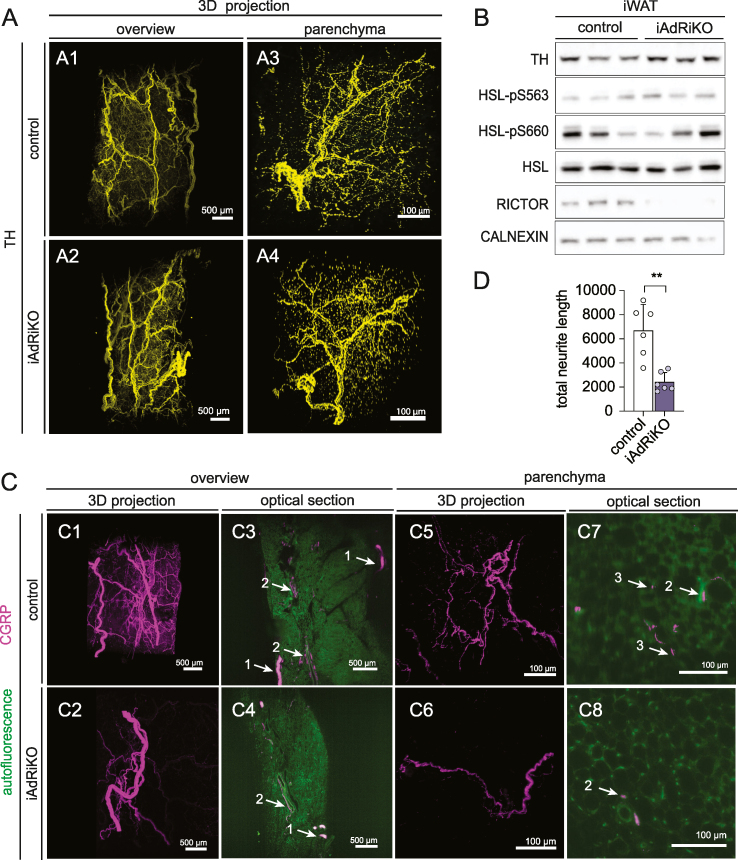
**Sensory but not sympathetic innervation is altered upon loss of adipose mTORC2**. (A) 2D representatives of a 3D reconstruction of inguinal WAT (iWAT) four weeks after tamoxifen treatment immunostained with tyrosine hydroxylase (TH; yellow). (A1-2) Low magnification projection of sympathetic neuronal network in control and iAdRiKO mice (N = 4; 5). Scale bar = 500 μm. (A3-4) High magnification projection of sympathetic neurons in iWAT parenchyma of control mice and iAdRiKO (N = 19; 10). Scale bar = 100 μm. (B) Immunoblot analysis of iWAT from control and iAdRiKO mice four weeks after tamoxifen treatment. Hormone-sensitive lipase (HSL). (n = 6; 6). (C) 2D representatives of a 3D reconstruction of iWAT four weeks after tamoxifen treatment immunostained with calcitonin gene-related peptide (CGRP; magenta). (C1-2) Low magnification projection of sensory neuronal network in control and iAdRiKO mice (N = 12; 19). Scale bar = 500 μm. (C3-4) Low magnification cross section of sensory neuronal network in control and iAdRiKO mice (N = 12; 19). Nerve bundle (1), innervation along blood vessel (2), tissue autofluorescence (green). Scale bar = 500 μm. (C5-6) High magnification projection of sensory neurons in iWAT parenchyma of control mice and iAdRiKO (N = 16; 11). Scale bar = 100 μm. (C7-8) High magnification cross section of neurons in control and iAdRiKO mice (N = 16; 11). Innervation along blood vessel (2), parenchymal innervation (3), tissue autofluorescence (green). Scale bar = 100 μm. (D) Quantification of the total neurite length of CGRP-positive neurons in iWAT parenchyma of control mice and iAdRiKO four weeks after tamoxifen treatment (N = 6).

**Supplementary Figure 3:**
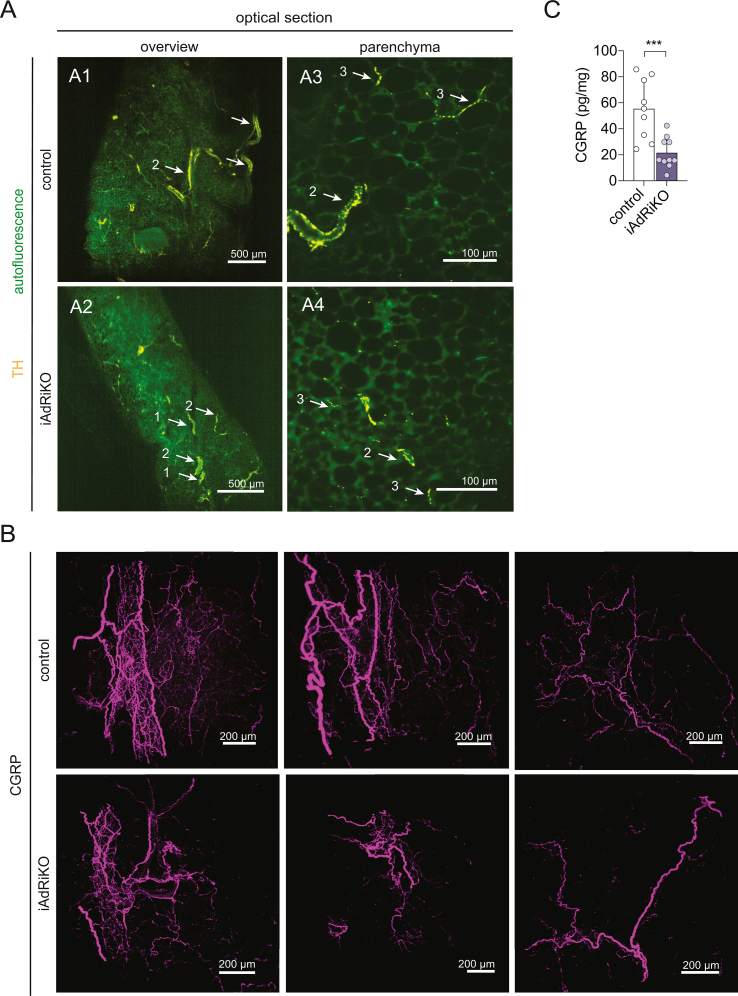
(A) Crosssections of inguinal WAT (iWAT) four weeks after tamoxifen treatment immunostained with tyrosine hydroxylase (TH; yellow). (A1-2) Low magnification cross sections of sympathetic neuronal network in control and iAdRiKO mice (N = **4; 5**). Nerve bundle (1), innervation around blood vessel (2), tissue autofluorescence (green). Scale bar = 500 μm. (A3-4) High magnification cross section of sympathetic neurons in iWAT parenchyma (3) of control and iAdRiKO mice (N = 19; 10). Scale bar = 100 μm. (B) High magnification projections of calcitonin gene-related peptide (CGRP)-positive sensory neurons (magenta) in iWAT of different control and iAdRiKO mice. Scale bar = 200 μm. (N = 13; 8, n = 3; 3). (C) Enzyme-linked immunosorbent assay (ELISA) for CGRP levels in iWAT of control and iAdRiKO mice (n = 9). Student's t-test, ∗∗∗p < 0.001.

To investigate the consequences of altered phosphorylation of synaptic proteins, as observed upon acute loss of adipose mTORC2, we examined neuronal innervation in iWAT four weeks after tamoxifen treatment. We first examined the sympathetic nervous system by visualizing TH-positive neurons in iWAT, using a modified whole-mount volumetric imaging protocol [34,43]. We combined the tissue preparation protocol described by Chi et al. [44] with a water-based clearing method [35] to enhance structure preservation and antibody compatibility. As described previously [43,44], we detected a dense network of TH-positive sympathetic neurons in nerve bundles that branched into smaller fibers running along blood vessels and into the parenchyma (Figure S3A). However, we found no change in abundance or morphology of TH-positive sympathetic neurons in iWAT of iAdRiKO mice, compared to controls (Figure 3A). In agreement with this result, immunoblot analyses showed no change in TH expression in iWAT of iAdRiKO mice (Figure 3B). Next, we analyzed the activity of sympathetic neurons in iWAT. TH-positive neurons release NE to stimulate β-adrenergic signaling in adipocytes, which promotes phosphorylation of hormone sensitive lipase (HSL) at S563 and S660 [45,46]. Immunoblot analyses revealed no difference in phosphorylation of S563 or S660 (Figure 3B), indicating similar sympathetic activity in iAdRiKO and control mice. Taken together, these findings suggest that the morphology and activity of the sympathetic nervous system are normal in iWAT of iAdRiKO mice.

We next examined the sensory nervous system in iWAT. Since the three-dimensional sensory neuronal network in iWAT was not previously described, we first examined CGRP-positive sensory neurons in control mice. Similar to TH-positive neurons, CGRP-positive sensory neurons innervated iWAT via large nerve bundles (Figure 3C). From the large nerve bundles, smaller nerve bundles branched off and ran along blood vessels into the tissue (Figure 3C). Using high magnification imaging, we detected single nerve fibers branching out into the parenchyma where sensory neurons were in close contact with adipocytes (Figure 3C).

**Figure 4:**
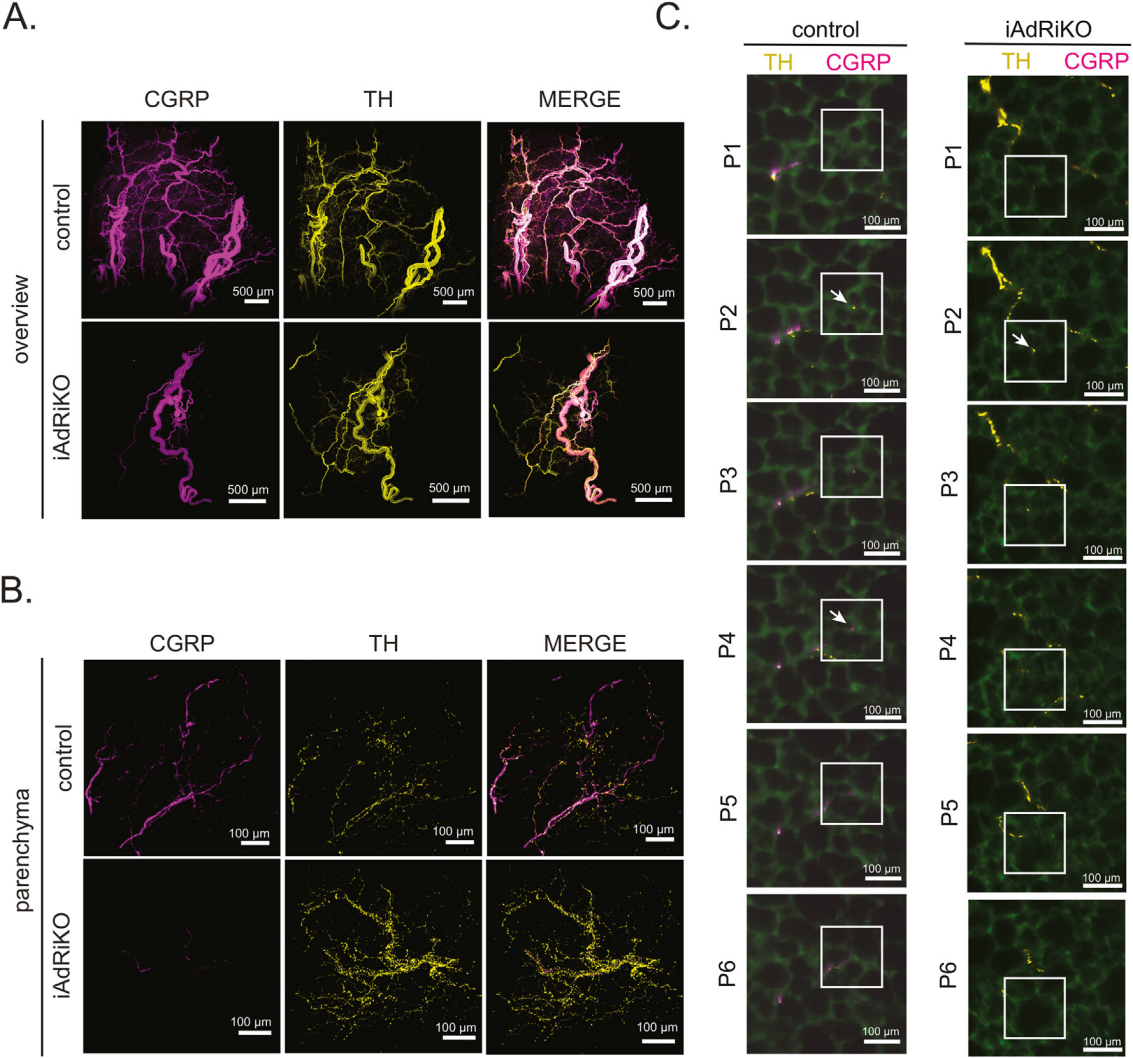
**Loss of adipose mTORC2 reduces sensory neurons in the proximity of adipocytes**. (A) Low magnification projection of inguinal WAT (iWAT) four weeks after tamoxifen treatment co-immunostained with tyrosine hydroxylase (TH, yellow) and calcitonin gene-related peptide (CGRP, magenta) (N = 15; 18). Scale bar = 500 μm. (B) High magnification projection of iWAT four weeks after tamoxifen treatment co-immunostained with TH (yellow) and CGRP (magenta) (N = 21; 22). Scale bar = 100 μm. (C) Subsequent sections (P1–P6; 6.48/6.28 μm interval, respectively) of control and iAdRiKO iWAT immunostained with TH (yellow) and CGRP (magenta) illustrating single nerve fibers innervating the periphery. Region of interest: Nerve ending and potential synapses (arrows). Tissue autofluorescence = green. Scale bar = 100 μm.

**Supplementary Figure 4:**
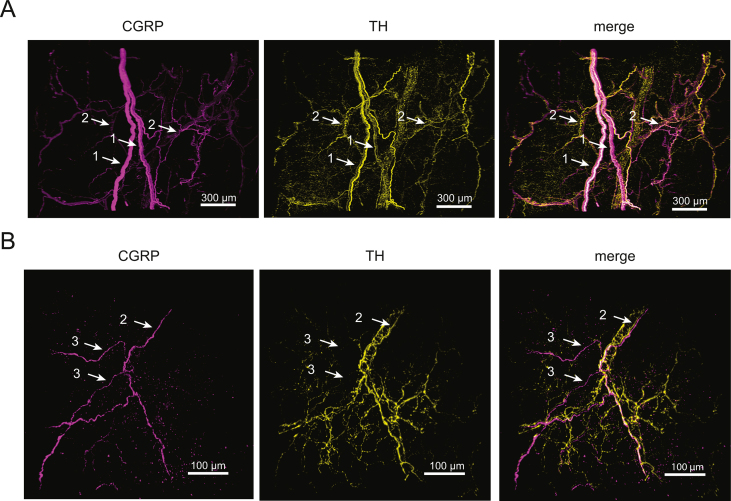
(A) 2D representative of a 3D reconstruction of an inguinal WAT (iWAT) section immunostained with calcitonin gene-related peptide (CGRP, magenta) and the tyrosine hydroxylase (TH, yellow) that illustrates how a large nerve bundle subdivides into smaller nerve bundles which in turn penetrate the tissue alongside blood vessels. Nerve bundle (1), innervation along blood vessel (2). Scale bar= 300 μm. (B) 2D representative of a 3D reconstruction of the iWAT parenchyma of both CGRP-positive (magenta) and the TH-positive (yellow) neuronal networks in iWAT. Innervation along blood vessel (2), parenchymal innervation (3). Scale bar= 100 μm.

**Supplementary Figure 5:**
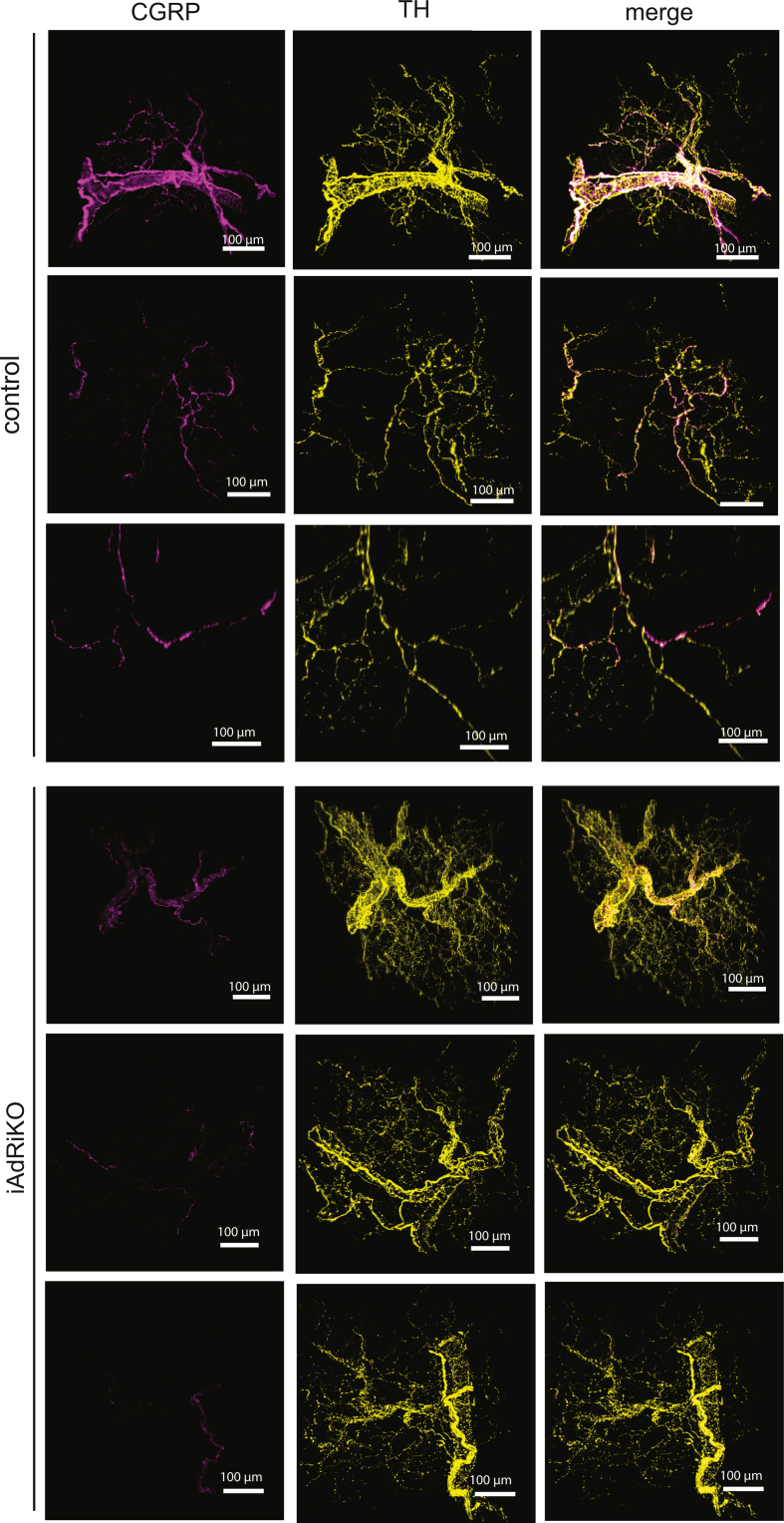
Representative of a 3D reconstruction of inguinal WAT sections from control and iAdRiKO mice immunostained with calcitonin gene-related peptide (CGRP; magenta) and tyrosine hydroxylase (TH; yellow) (N=21;22). Scale bar= 100 μm.

Next, we analyzed the sensory neuronal network in iWAT of iAdRiKO mice. CGRP-positive sensory neurons were present in large and small nerve bundles similar to control mice (Figures 3C and S3B). However, unlike control mice, we did not detect CGRP-positive neurons in the parenchyma, indicating loss of arborization of sensory neurons in iWAT of iAdRiKO mice (Figures 3C and S3B, Movie 1, Movie 2). Quantification of overall neurite length confirmed the reduction of sensory innervation in iWAT lacking mTORC2 (Figure 3D). Furthermore, an enzyme-linked immunosorbent assay (ELISA) revealed reduced levels of CGRP in iAdRiKO iWAT (Figure S3C). To further investigate the loss of CGRP-positive sensory neurons in iWAT, we co-stained and visualized TH- and CGRP-positive neurons in whole iWAT depots collected from iAdRiKO and control mice. We discovered that TH- and CGRP-positive neurons were present in the same larger and smaller nerve bundles and ran alongside each other into the tissue (Figures 4A and S4A–B, Movie 3. However, the TH-positive sympathetic neuronal network was much more dense compared to the sensory neuronal network, particularly in the parenchyma, in both iAdRiKO and control mice. Confirming the above observations, sensory neurons were not detected in the parenchyma of iAdRiKO iWAT, while sympathetic neurons were detected (Figures 4B–C and S5, Movie 4, Movie 5). Taken together, these findings suggest that adipose mTORC2 is required for arborization of CGRP-positive sensory neurons, but not of sympathetic neurons, in iWAT.

**Supplementary Figure 6:**
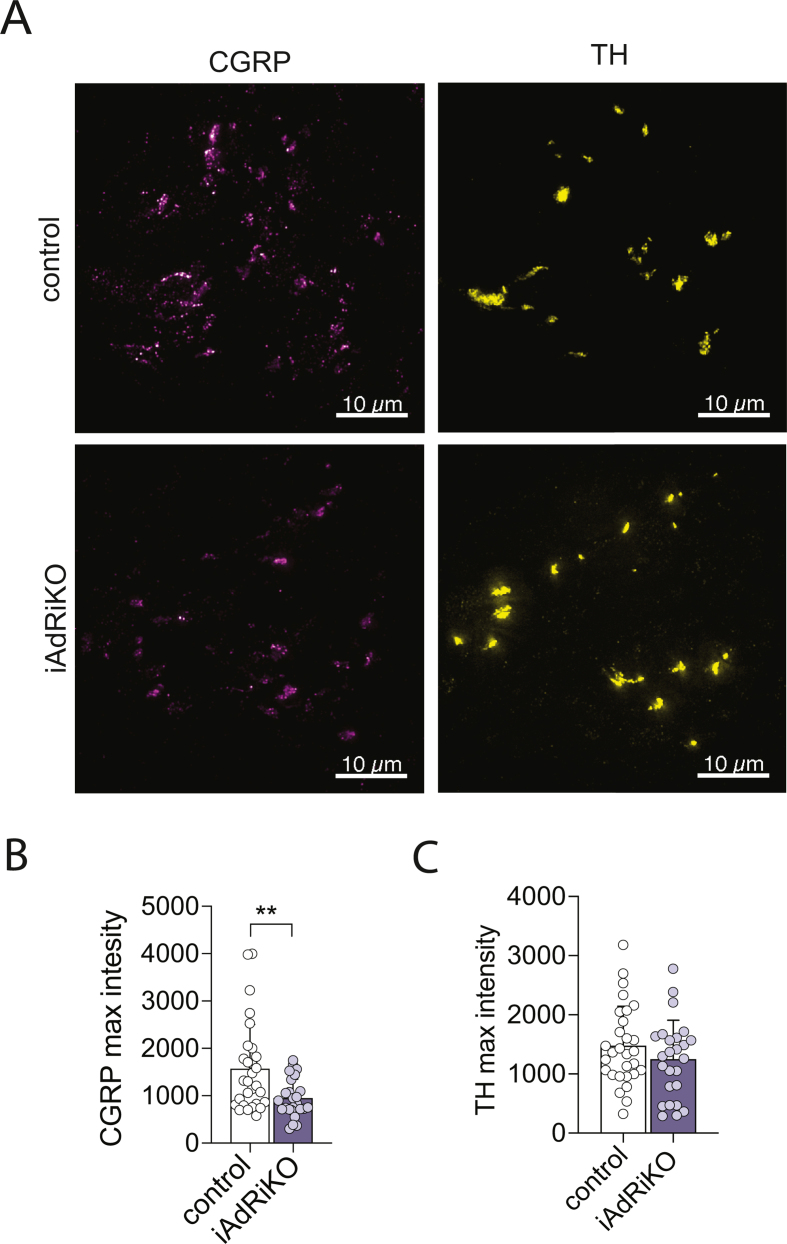
(A) Representative image of a large nerve bundle in control and iAdRiKO iWAT paraffine sections immunostained with CGRP (magenta) and tyrosine hydroxylase (TH, yellow) (N= 29;25). Scale bar= 10 μm. (B) Quantification of maximal intensity of CGRP-immunostaining in large nerve bundles of iWAT of control and iAdRiKO mice (N= 29;25). Student’s t-test, ∗∗p<0.01. (C) Quantification of maximal intensity of TH-immunostaining in large nerve bundles of iWAT of control and iAdRiKO mice (N= 29;25).

We noted that the intensity of CGRP staining was slightly reduced in the main nerve bundles of iAdRiKO iWAT (Figure 3C). To confirm this observation, we performed co-staining of TH and CGRP in sectioned iWAT (Figure S6A). The number of CGRP-positive single fibers was similar in nerve bundles of iAdRiKO and control mice. However, the intensity of CGRP per fiber was reduced 40% in iAdRiKO iWAT (Figure S6A–B). Consistent with whole-mount imaging (Figure 3A), we detected no change in TH intensity in sectioned iWAT of iAdRiKO mice (Figure S6A and S6C). The reduction of CGRP in single nerve fibers, in addition to loss of arborization, may indicate a loss in sensory activity.

Supplementary video related to this article can be found at https://doi.org/10.1016/j.molmet.2022.101580

The following is/are the supplementary data related to this article:

Movie 1 Sensory neuronal network in control mouse. Calcitonin gene-related peptide (CGRP)-positive neuronal network (magenta) in the inguinal WAT parenchyma of a control mouse Movie 1

Movie 2 Sensory neuronal network in iAdRiKO mouse. CGRP- positive neuronal network (magenta) in the inguinal WAT parenchyma of an iAdRiKO mouse Movie 2

Movie 3 Overview neuronal innervation. Overview of both CGRP(magenta)- and tyrosine hydroxylase (TH; yellow)-positive neuronal networks in inguinal WAT of a control mouse. Movie 3

Movie 4 Neuronal networks in control mouse. CGRP(magenta)- and TH(yellow)-positive neuronal networks in the inguinal WAT parenchyma of a control mouse. Movie 4

Movie 5 Neuronal networks in iAdRiKO mouse. CGRP(magenta)- and TH(yellow)- positive neuronal networks in the inguinal WAT parenchyma of an iAdRiKO mouse. Movie 5

### 3.4 Adipose mTORC2 stabilizes sensory neurons

**Figure 5:**
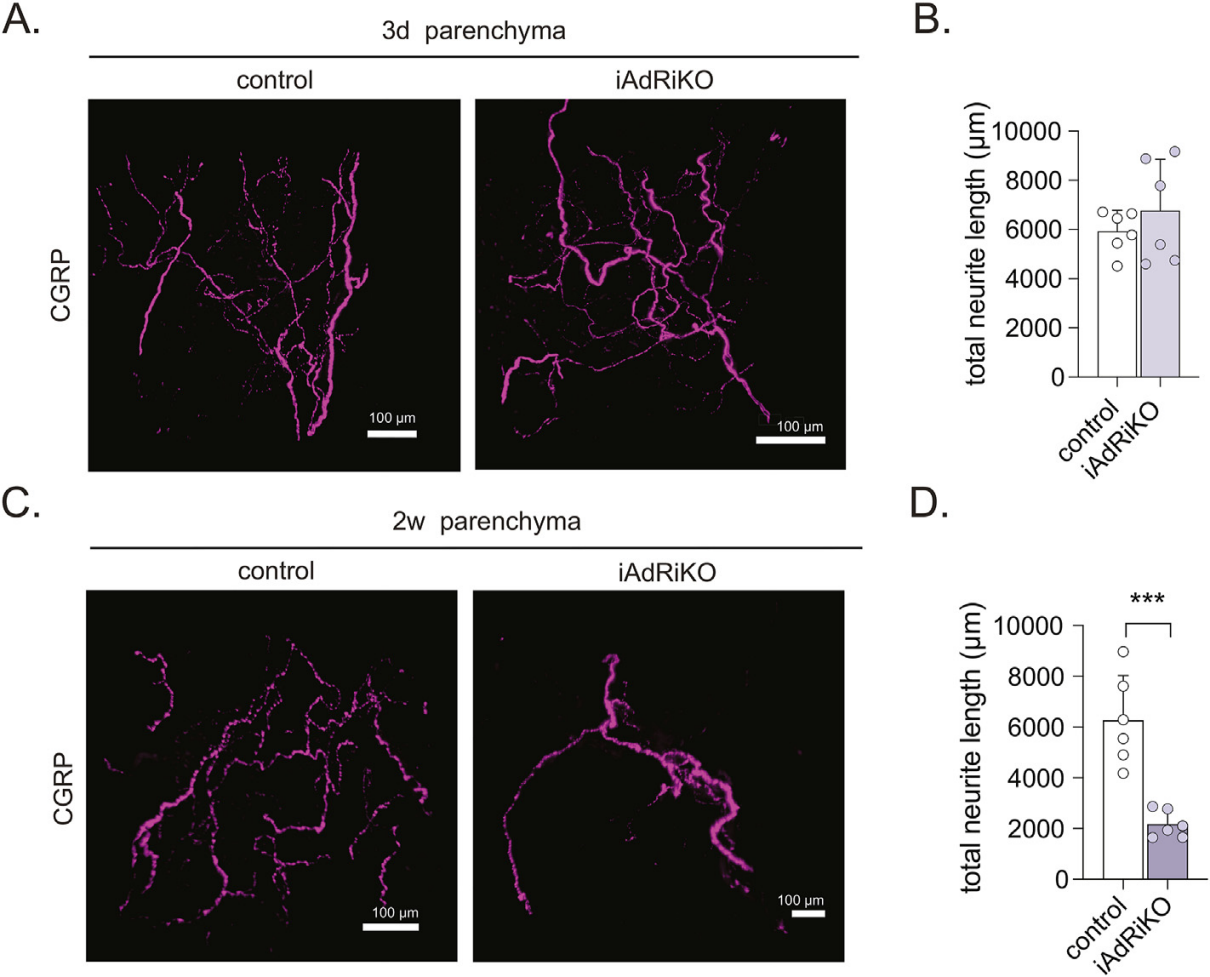
**Adipose mTORC2 maintains sensory neurons**. (A–B) High magnification projection (A) and quantification of the total neurite length (B) of CGRP-positive neurons in iWAT parenchyma of control mice and iAdRiKO three days after tamoxifen treatment (N = 6). Scale bar = 100 μm. (C–D) High magnification projection (C) and quantification of the total neurite length (D) of CGRP-positive neurons in iWAT parenchyma of control mice and iAdRiKO two weeks after tamoxifen treatment (N = 6). Scale bar = 100 μm; Student's t-test, ∗∗∗p < 0.001.

The above showed that sensory neurons are missing four weeks after mTORC2 ablation. We next investigated whether adipose mTORC2 is required to establish or maintain sensory neurons by examining the sensory neuronal network in iWAT at three days and two weeks after tamoxifen treatment. The sensory neuronal network was fully developed at the beginning of the post-tamoxifen time course (∼7 week old mice) in control and iAdRiKO iWAT. The sensory neuronal network was also intact three days after tamoxifen treatment (Figure 5A–B). However, arborization of sensory neurons was significantly diminished two weeks after mTORC2 ablation (Figure 5C–D). These data indicate rapid pruning of sensory neurons upon loss of adipose mTORC2. Thus, adipose mTORC2 is required for the stability of sensory neurons in WAT.

### 3.5 GAP43 is a marker for sensory neurons in adipose tissue

**Figure 6:**
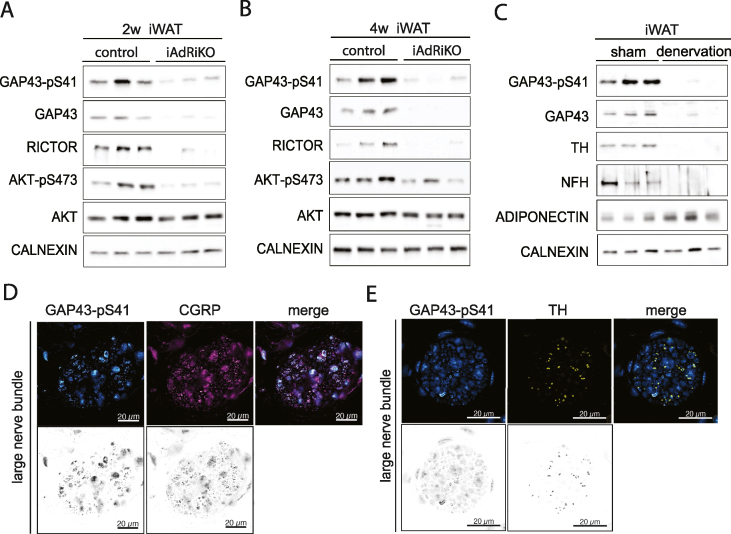
**GAP43 expression is downregulated in CGRP-positive neurons upon loss of adipose mTORC2**. (A) Immunoblot analysis of inguinal WAT (iWAT) tissue from control and iAdRiKO mice two weeks after tamoxifen treatment. (n = 6; 6). (B) Immunoblot analysis of iWAT tissue from control and iAdRiKO mice four weeks after tamoxifen treatment. (n = 6; 6). (C) Immunoblot analysis of surgically denervated iWAT depot (denervation) compared to iWAT depot from sham-operated mice (sham). Neurofilament heavy polypeptide (NFH). (n = 5; 5). (D) Representative image of a large nerve bundle in iWAT of control mice immunostained with growth-associated protein 43 (GAP43)-pS41 and calcitonin gene-related peptide (CGRP). (N = 11; 9). (E) Representative image of a large nerve bundle in iWAT of control mice immunostained with GAP43-pS41 and tyrosine hydroxylase (TH). (N = 19; 11).

We next searched for a marker for sensory innervation in iAdRiKO WAT. GAP43 is a neuronal protein that promotes neuronal growth and plasticity in the CNS [47] and has been shown to be downregulated in iWAT of *ob/ob* mice, a mouse model for type II diabetes [48]. Immunoblot analysis of total and phosphorylated (pS41) GAP43 showed a strong decrease in GAP43 expression in iWAT lacking mTORC2, at two and four weeks after tamoxifen treatment (Figure 6A–B). Since WAT is a heterogenous tissue, we next examined whether GAP43 is expressed only in neurons. We determined GAP43 expression in surgically denervated iWAT, observing that GAP43 expression was indeed lost upon denervation (Figure 6C). These data suggest that GAP43 is expressed only in neurons and thus the loss of GAP43 observed in iWAT of iAdRiKO is due to loss in the nervous system.

Previous reports showed that GAP43 can be expressed in both sensory and sympathetic neurons [49,50]. To address which class of neurons express GAP43 in iWAT, we visualized GAP43 in TH- or CGRP-positive neurons in large nerve bundles in sectioned iWAT. We note that we visualized GAP43 with antibody against phosphorylated GAP43 because antibody against total GAP43 did not serve as an immunostaining reagent. We found that the GAP43-pS41 signal correlated with CGRP-positive fibers, but not with TH-positive fibers, indicating that GAP43 is expressed specifically in sensory neurons in iWAT (Figure 6D–E). We conclude that GAP43 is a useful marker to examine sensory neurons in WAT.

## 4 Discussion

In this study, we provide insights on the morphology, role and regulation of the sensory nervous system in WAT. Taking advantage of recent progress in whole-mount three-dimensional imaging, we show that sensory neurons are present in central nerve bundles, along blood vessels and in the parenchyma of WAT. Furthermore, we discovered that loss of mTORC2 signaling in adipocytes decreases arborization of sensory neurons without affecting sympathetic innervation or activity. Our findings suggest that adipose mTORC2 stabilizes CGRP-positive sensory neurons in WAT, thereby supporting adipocyte-to-CNS communication.

The morphology of the sensory nervous system in WAT was not described previously. We show that CGRP-positive sensory neurons, like TH-positive sympathetic neurons, innervate the parenchyma of WAT. We note that the sensory nervous system forms a less dense network compared to the sympathetic nervous system. Nevertheless, sensory neurons appear to contact adipocytes (Figure 4C). The nature of these connections is still unknown; sensory neurons may form stable synapses with adipocytes or terminate as free nerve endings. However, the close proximity of adipocytes and sensory neurons provides morphological evidence for adipocyte-to-CNS communication via the sensory nervous system.

The role of sensory neurons in WAT is poorly understood. It has been proposed that sensory neurons may directly communicate the metabolic state of WAT to CNS [[13], [14], [15]]. Our data provide evidence that this communication is disrupted upon ablation of adipose mTORC2, a critical regulator of energy uptake and storage in WAT [23,25]. We found that reduction of sensory innervation in WAT coincides with systemic insulin resistance. It remains to be determined whether there is indeed a causal relationship between the observed changes in CGRP-positive neurons in WAT and wholebody energy homeostasis.

How does adipose mTORC2 stabilize CGRP-positive sensory neurons in WAT? Our phosphoproteomic analysis revealed that phosphorylation of both membrane-associated proteins and cell–cell adhesion proteins was altered upon loss of adipose mTORC2 (Figure 2F). Thus, loss of mTORC2 in adipocytes may disrupt the postsynaptic membrane, thereby destabilizing synapses. Alternatively, a secreted factor from adipocytes may promote stability of CGRP-positive sensory neurons. It has been suggested that sensory neurons respond to secreted free fatty acids [15,51]. Since mTORC2 promotes fatty acid synthesis [23,52], loss of mTORC2 may decrease production of bioactive free fatty acid species by adipocytes, which may in turn decrease arborization of sensory neurons. Further studies are required to determine the molecular mechanism underlying adipose mTORC2-mediated regulation of CGRP-positive sensory neurons in iWAT.

Degeneration of sensory neurons has been observed in diabetic patients, a condition known as diabetes-induced neuropathy [53]. However, the underlying mechanism(s) is poorly understood. Blaszkiewicz et al. reported that GAP43-positive neurons are lost in iWAT in genetically obese (*ob/ob*) mice [48]. Since a decrease in mTORC2 has been shown in omental WAT of obese patients [26], it will also be of interest to investigate a possible correlation between loss of CGRP-positive neurons and reduced mTORC2 activity in WAT as a potential mechanism of diabetes-induced neuropathy.

## Author contributions

ICF, MS, and MNH designed the project and the experiments. ICF, DW and MS conducted the experiments with contributions from DR (proteomics), MC (proteomic analysis) and WH (clearing method). ICF, MS and MNH wrote the manuscript.

## Data Availability

Data will be made available on request.

## Appendix A

### Supplementary Materials and Methods

#### Sample preparation for proteomics and phosphoproteomics

Tissues were pulverized and homogenized in lysis buffer containing 100 mM Tris–HCl pH7.5, 2 mM EDTA, 2 mM EGTA, 150 mM NaCl, 1% Triton X-100, cOmplete inhibitor cocktail (Roche) and PhosSTOP (Roche). Samples were lysed by polytron followed by ultrasonication. Lysates was further cleared from debris and excessive amount of lipids by centrifugation (2 × 14′000 g, 10min). Proteins were precipitated by trichloroacetic acid (Sigma), washed with cold acetone and resuspended in buffer containing 1.6 M urea, 0.1 M ammonium bicarbonate and 5 mM TCEP. Proteins were alkylated with 10 mM chloroacetamide and digested with sequencing-grade modified trypsin (enzyme/protein ratio 1:50) overnight. After acidification with 5% TFA, peptides were desalted using C18 reverse-phase spin columns (Macrospin, Harvard Apparatus) according to the manufacturer's instructions, dried under vacuum and stored at −20 °C until further use.

For TMT-labelling, 25 μg of peptides per sample were labeled with isobaric tandem mass tags (TMT10plex, Thermo Fisher Scientific) as described previously. In brief, peptides were resuspended in 20 μl labeling buffer (2 M urea, 0.2 M HEPES, pH 8.3) and a peptide calibration mixture consisting of six digested standard proteins mixed in different amounts was spiked into each sample before TMT reagents were added, followed by a 1 h incubation at 25 °C shaking at 500 rpm. To quench the labelling reaction, aqueous 1.5 M hydroxylamine solution was added and samples were incubated for another 5 min at 25 °C shaking at 500 rpm followed by pooling of all samples. The pH of the sample pool was increased to 11.9 by adding 1 M phosphate buffer (pH 12) and incubated for 20 min at 25 °C shaking at 500 rpm to remove TMT labels linked to peptide hydroxyl groups. Subsequently, the reaction was stopped by adding 2 M hydrochloric acid until a pH < 2 was reached. Finally, peptide samples were further acidified using 5% TFA, desalted using Sep-Pak Vac 1cc (50 mg) C18 cartridges (Waters) according to the manufacturer's instructions and dried under vacuum.

#### Proteomics

TMT-labeled peptides were fractionated by high-pH reversed phase separation using a XBridge Peptide BEH C18 column (3.5 μm, 130 Å, 1 mm × 150 mm, Waters) on an Agilent 1260 Infinity HPLC system. Peptides were loaded on column in buffer A (20 mM ammonium formate in water, pH 10) and eluted using a two-step linear gradient from 2% to 10% in 5 min and then to 50% buffer B (20 mM ammonium formate in 90% acetonitrile, pH 10) over 55 min at a flow rate of 42 μl/min. Elution of peptides was monitored with a UV detector (215 nm, 254 nm) and a total of 36 fractions were collected, pooled into 12 fractions using a post-concatenation strategy as previously described and dried under vacuum. Dried peptides were resuspended in 0.1% aqueous formic acid and subjected to LC–MS/MS analysis using a Q Exactive HF Mass Spectrometer fitted with an EASY-nLC (both Thermo Fisher Scientific) and a custom-made column heater set to 60 °C. Peptides were resolved using a RP-HPLC column (75 μm × 30 cm) packed in-house with C18 resin (ReproSil-Pur C18–AQ, 1.9 μm resin; Dr. Maisch GmbH) at a flow rate of 0.2 μl/min. The following gradient was used for peptide separation: from 5% B to 15% B over 19 min to 30% B over 80 min to 45% B over 21 min to 95% B over 2 min followed by 18 min at 95% B. Buffer A was 0.1% formic acid in water and buffer B was 80% acetonitrile, 0.1% formic acid in water. The Q Exactive HF mass spectrometer was operated in DDA mode with a total cycle time of approximately 1 s. Each MS1 scan was followed by high-collision-dissociation (HCD) of the 10 most abundant precursor ions with dynamic exclusion set to 30 s. For MS1, 3e6 ions were accumulated in the Orbitrap over a maximum time of 100 ms and scanned at a resolution of 120,000 FWHM (at 200 *m/z*). MS2 scans were acquired at a target setting of 1e5 ions, maximum accumulation time of 100 ms and a resolution of 30,000 FWHM (at 200 *m/z*). Singly charged ions and ions with unassigned charge state were excluded from triggering MS2 events. The normalized collision energy was set to 35%, the mass isolation window was set to 1.1 *m/z* and one microscan was acquired for each spectrum. The acquired raw-files were converted to the mascot generic file (mgf) format using the msconvert tool (part of ProteoWizard, version 3.0.4624 (2013-6-3)) and searched using MASCOT against a murine database (consisting of 34026 forward and reverse protein sequences downloaded from Uniprot on 20190129), the six calibration mix proteins and 392 commonly observed contaminants. The precursor ion tolerance was set to 10 ppm and fragment ion tolerance was set to 0.02 Da. The search criteria were set as follows: full tryptic specificity was required (cleavage after lysine or arginine residues unless followed by proline), 3 missed cleavages were allowed, carbamidomethylation (C) and TMT6plex (K and peptide N-terminus) were set as fixed modification and oxidation (M) as a variable modification. Next, the database search results were imported into the Scaffold Q+ software (version 4.3.2, Proteome Software Inc., Portland, OR) and the protein false identification rate was set to 1% based on the number of decoy hits. Proteins that contained similar peptides and could not be differentiated based on MS/MS analysis alone were grouped parsimoniously. Proteins sharing significant peptide evidence were grouped into clusters. Acquired reporter ion intensities in the experiments were employed for automated quantification and statistical analysis using a modified version of our in-house developed SafeQuant R script. This analysis included adjustment of reporter ion intensities, global data normalization by equalizing the total reporter ion intensity across all channels, summation of reporter ion intensities per protein and channel, calculation of protein abundance ratios and testing for differential abundance using empirical Bayes moderated t-statistics. To meet additional assumptions (normality and homoscedasticity) underlying the use of linear regression models and t-Tests, MS-intensity signals were transformed from the linear to the log-scale. All proteins detected are presented in Supplementary Table 2. Finally, significantly deregulated proteins were defined as log2 (fold change) > 0.6 or log2 (fold change) < −0.6, p-value < 0.01.

#### Phosphoproteomics

Peptide samples were enriched for phosphorylated peptides using Fe(III)-IMAC cartridges on an AssayMAP Bravo platform as described. Unmodified peptides (“flowthrough”) were subsequently used for TMT analysis. Phospho-enriched peptides were resuspended in 0.1% aqueous formic acid and subjected to LC–MS/MS analysis using an Orbitrap Fusion Lumos Mass Spectrometer fitted with an EASY-nLC 1200 (both Thermo Fisher Scientific) and a custom-made column heater set to 60 °C. Peptides were resolved using a RP-HPLC column (75 μm × 37 cm) packed in-house with C18 resin (ReproSil-Pur C18–AQ, 1.9 μm resin; Dr. Maisch GmbH) at a flow rate of 0.2 μl/min. The following gradient was used for peptide separation: from 5% B to 8% B over 5 min to 20% B over 45 min to 25% B over 15 min to 30% B over 10 min to 35% B over 7 min to 42% B over 5 min to 50% B over 3min to 95% B over 2 min followed by 18 min at 95% B. Buffer A was 0.1% formic acid in water and buffer B was 80% acetonitrile, 0.1% formic acid in water. The Orbitrap Fusion Lumos mass spectrometer was operated in DDA mode with a cycle time of 3 s between master scans. Each master scan was acquired in the Orbitrap at a resolution of 120,000 FWHM (at 200 *m/z*) and a scan range from 375 to 1600 *m/z* followed by MS2 scans of the most intense precursors in the Orbitrap at a resolution of 30,000 FWHM (at 200 *m/z*) with isolation width of the quadrupole set to 1.4 *m/z*. Maximum ion injection time was set to 50 ms (MS1) and 54 ms (MS2) with an AGC target set to 1e6 and 5e4, respectively. Only peptides with charge state 2–5 were included in the analysis. Monoisotopic precursor selection (MIPS) was set to Peptide, and the Intensity Threshold was set to 2.5e4. Peptides were fragmented by HCD (Higher-energy collisional dissociation) with collision energy set to 30%, and one microscan was acquired for each spectrum. The dynamic exclusion duration was set to 30 s. The acquired raw-files were imported into the Progenesis QI software (v2.0, Nonlinear Dynamics Limited), which was used to extract peptide precursor ion intensities across all samples applying the default parameters. The generated mgf-file was searched using MASCOT against a murine database (consisting of 34026 forward and reverse protein sequences downloaded from Uniprot on 2019-01-29) and 392 commonly observed contaminants using the following search criteria: full tryptic specificity was required (cleavage after lysine or arginine residues, unless followed by proline); 3 missed cleavages were allowed; carbamidomethylation (C) was set as fixed modification; oxidation (M) and phosphorylation (STY) were applied as variable modifications; mass tolerance of 10 ppm (precursor) and 0.02 Da (fragments). The database search results were filtered using the ion score to set the false discovery rate (FDR) to 1% on the peptide and protein level, respectively, based on the number of reverse protein sequence hits in the datasets. Exported peptide intensities were normalized based on the protein regulations observed in the corresponding TMT experiment in order to account for changes in protein abundance. Only peptides corresponding to proteins, which were regulated significantly with a p value ≤ 1% in the TMT analysis were normalized. Quantitative analysis results from label-free quantification were processed using the SafeQuant R package v.2.3.2 (https://github.com/eahrne/SafeQuant/) to obtain peptide relative abundances. This analysis included global data normalization by equalizing the total peak/reporter areas across all LC-MS runs, data imputation using the knn algorithm, summation of peak areas per peptide and LC-MS/MS run, followed by calculation of peptide abundance ratios. Only isoform specific peptide ion signals were considered for quantification. The summarized peptide expression values were used for statistical testing of between condition differentially abundant peptides. Here, empirical Bayes moderated t-Tests were applied, as implemented in the R/Bioconductor limma package were used. All LC-MS analysis runs were acquired from independent biological samples. To meet additional assumptions (normality and homoscedasticity) underlying the use of linear regression models and t-Tests, MS-intensity signals were transformed from the linear to the log-scale. All proteins detected are presented in Supplementary Table 2. Finally, significantly deregulated were selected by a calculated p-value < 0.01.

#### Further statistical analysis for proteomics and phosphoprotemics

Using the software Perseus we performed PCA after Lg2 transformation of the pre normalized Intensity data derived as described in section phosphoproteomics. To generate hierarchical clustering with z-scored data, we used Spearman correlation for the distance of both trees, leaving the other parameters as default. For the volcano-plots, we used the scatter plot function within Excel. Log2 fold changes and p-values were generated with SafeQuant and Limma R packages as described in sections phosphoroteomics and proteomics.

#### Pathway enrichment analysis

Pathway enrichment analysis for proteins with altered phosphorylation sites (p-value < 0.01) between iAdRiKO and control mice was performed by the Database for Annotation, Visualization and Integrated discovery (DAVID) v6.8 (https://david.ncifcrf.gov/). Settings for DAVID were default. The background list consisted of all proteins detected in the phosphoproteome data set (Supplementary Table 2). The full analysis can be found in Supplementary Table 3.

## Appendix A Supplementary data

Supplementary Table S1 (A) Proteome data set from inguinal WAT (iWAT) of iAdRiKO and control mice three days after tamoxifen treatment. (B) Phosphoproteomics data set from iWAT of iAdRiKO and control mice three days after tamoxifen treatment. Significant hits are highlighted in yellow (p < 0.01). Supplementary Table S1
